# Therapeutic potential of mesenchymal stromal/stem cells in critical‐care patients with systemic inflammatory response syndrome

**DOI:** 10.1002/ctm2.1163

**Published:** 2023-01-01

**Authors:** Gerardo‐Javier Martí‐Chillón, Sandra Muntión, Silvia Preciado, Lika Osugui, Almudena Navarro‐Bailón, Javier González‐Robledo, Víctor Sagredo, Juan F. Blanco, Fermín Sánchez‐Guijo

**Affiliations:** ^1^ IBSAL‐University Hospital of Salamanca Salamanca Spain; ^2^ Department of Medicine University of Salamanca Salamanca Spain; ^3^ RICORS TERAV ISCIII Madrid Spain; ^4^ Regenerative Medicine and Cellular Therapy Network Center of Castilla y León Salamanca Spain; ^5^ Department of Surgery University of Salamanca Salamanca Spain

**Keywords:** cellular therapy, immunomodulation, intensive care, mesenchymal stromal/stem cells, MSCs, sepsis, SIRS, systemic inflammatory response syndrome

## Abstract

**Background:**

Despite notable advances in the support and treatment of patients admitted to the intensive care unit (ICU), the management of those who develop a systemic inflammatory response syndrome (SIRS) still constitutes an unmet medical need.

**Main body:**

Both the initial injury (trauma, pancreatitis, infections) and the derived uncontrolled response promote a hyperinflammatory status that leads to systemic hypotension, tissue hypoperfusion and multiple organ failure. Mesenchymal stromal/stem cells (MSCs) are emerging as a potential therapy for severe ICU patients due to their potent immunomodulatory, anti‐inflammatory, regenerative and systemic homeostasis‐regulating properties. MSCs have demonstrated clinical benefits in several inflammatory‐based diseases, but their role in SIRS needs to be further explored.

**Conclusion:**

In the current review, after briefly overviewing SIRS physiopathology, we explore the potential mechanisms why MSC therapy could aid in the recovery of this condition and the pre‐clinical and early clinical evidence generated to date.

## Graphical abstract

- Mesenchymal stromal/stem cells therapeutic potential for SIRS.
- MSCs may control the exacerbated immune reaction after a critical insult (e.g. polytrauma, severe infections, burns and pancreatitis) at local and systemic levels.
- MSCs can exert organ protection and tissue regeneration properties through direct and remote mechanisms.
- MSCs are able to promote homeostasis recovery.

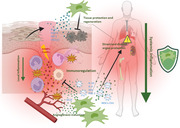

## 1 INTRODUCTION

Systemic inflammatory response syndrome (SIRS) consists of an exacerbated immune reaction following severe injuries such as trauma, pancreatitis or infections (the latter known as sepsis).^1^, ^2^ This acute hyperinflammatory response alters the systemic homeostasis leading to shock and multiorgan dysfunction.^3^ Added to the initial injury, SIRS aggravates the clinical condition and compromises the survival of patients admitted to the intensive care unit (ICU).

In contrast to patients without SIRS, patients who develop it are more likely to be admitted to the ICU, to require a higher level of care and to experience a higher mortality rate at 28 days.^4^, ^5^, ^6^ Sepsis is the primary cause of death from infection, which mortality reaches levels of 25%–30% and increases with the shock and multiorgan failure progression.^1^, ^7^ This makes SIRS be among the most common causes of mortality.^8^

After the initial injury, patient stabilization (haemodynamic and respiratory parameters) is crucial for early survival. Drugs administered and ICU supporting devices are employed depending on the symptoms and the clinical evolution of the patient (fluids, vasopressors, antibiotics, transfusion, mechanical ventilation etc.). The use of corticosteroids remains controversial in SIRS/sepsis due to the lack of evidence of their benefit.^2^, ^9^ Furthermore, diverse adverse events in specific conditions have been described with the use of corticosteroids, including hyperglycaemia, hypernatremia gastrointestinal bleeding, muscular weakness and infections.^10^ Following the idea to control the exacerbated inflammatory response during SIRS, some strategies have been proposed (e.g. prostaglandin E1, anti‐CD18 monoclonal antibodies and aselizumab).^2^ However, these strategies do not show improvement in patient outcome after comparing to standard care,^2^ and this fact supports the need to exploring novel therapeutic strategies, including cellular therapy. Despite advances in clinical and therapeutic approaches, a potential treatment for SIRS that could contribute to diminish the massive inflammatory processes and the tissue damage is still lacking.

In this setting, mesenchymal stromal/stem cells (MSCs) are a potentially attractive tool.^1^, ^11^ MSCs have demonstrated significant benefit in a number of inflammatory and immune‐based conditions, including graft‐versus‐host disease (GVHD) after hematopoietic stem cell transplantation and Crohn's disease (CD), where some advanced‐therapy medical products (ATMP) based on allogeneic mesenchymal stem cells have been approved in several countries after favourable outcome in clinical trials.^12^ Considering the anti‐inflammatory, immunomodulatory and regenerative mechanisms of action of MSCs, it is attractive to explore their clinical potential in inflammatory diseases, including SIRS. Moreover, in the case of SIRS, the role of massive inflammation in the pathophysiological process and in the unfavourable evolution is well known, and this is a clinical situation where cells may display a more relevant role, as has been observed at the pre‐clinical level and as is already being tested in clinical trials, as indicated in the corresponding section of this review.

## 2 PHYSIOPATHOLOGY OF SIRS

Although SIRS is a complex and heterogeneous inflammatory reaction, some common patterns are shared in most cases, independently from the initial injury. SIRS is triggered by the release of signals known as danger‐associated molecule patterns (DAMPs) from affected tissues (high‐mobility group box protein 1 – HMGB1, mitochondrial and intracellular components) or by the detection of pathogen‐derived molecules (lipopolysaccharide – LPS) by pattern‐recognition receptors from resident immune cells.^13^ This initial trigger activates immune cells and induces the secretion of multiple cellular mediators. This escalation of excessive signalling evolves into a cytokine storm that activates a massive mobilization of immune cells. Added to the inflammatory response, the alteration of the vascular endothelium integrity induces a state of hypotension and tissue hypoperfusion. These phenomena alter the homeostasis and integrity of whole‐body tissues that can lead to a multiple organ dysfunction syndrome (MODS).^14^, ^15^

The excessive release of cytotoxic immune products (reactive oxygen species – ROS, proteases), the unspecific activation and the inflammatory conditions lead to endothelial dysfunction.^16^ The endothelium has a critical role in SIRS patients as it is the key regulator of the coagulation balance, vascular tone, capillary permeability and specific leukocyte transendothelial migration. As previously mentioned, endothelium dysfunction increases uncontrolled fluidic extravasation promoting hypovolaemia, vasodilatation and capillary leakage.^14^ Furthermore, the microcirculation is affected by the capillary obstruction due to alterations in the coagulation endothelial‐dependent equilibrium and the uncontrolled release of pro‐coagulant factors, favouring the development of disseminated intravascular coagulation.^17^ This potential complication promotes emboli formation that hampers capillary blood supply to tissues. All these conditions alter oxygen and metabolic product exchanges, leading to hypoxia, ischaemia and tissue damage. Finally, the unspecific immune cell migration and overactivity further deteriorate organs and tissues not initially involved in the first injury, like lung (acute lung injury – ALI, acute respiratory distress syndrome – ARDS), kidney (acute kidney injury – AKI), heart or liver.^18^, ^19^

## 3 MESENCHYMAL STROMAL/STEM CELLS

MSCs are multipotent non‐hematopoietic cells described initially in bone marrow (BM) but present in almost all tissues and organs, which are involved in microenvironment regulation, extracellular matrix formation and tissue homeostasis maintenance.^20^ MSCs favour cell survival, stress reduction and tissue and organ protection.^21^

The investigation of MSCs as a therapeutic agent (considered an ATMP by the regulatory agencies) has increased in a broad range of diseases in part due to their availability from different sources (BM, adipose tissue, Wharton's jelly, umbilical cord etc.), ex vivo expansion, their biological properties and their low immunogenicity.^22^, ^23^ It is possible to obtain high amounts of MSCs ex vivo, allowing large‐scale production for autologous and allogenic therapy.^24^ It has been suggested that MSCs may vary in their differentiation and immunomodulatory potential depending on their source.^25^, ^26^ Although BM‐ and AT‐derived MSCs are by far the most used sources, UC‐MSCs have shown large‐scale expansion and longer retardation of senescence than adult tissue sources. In addition, some reports have shown that AT‐MSCs have higher immunomodulatory potential than BM‐MSCs.^26^ Although most of the pre‐clinical studies in septic models have employed BM‐MSCs (60%) followed by AT‐MSCs and UC‐MSCs,^27^ there is no definitive clue on the differences between cell sources for the treatment of SIRS or sepsis. Further research is required to determine in which strategy (tissue source, dose) is the most appropriate for each specific disease, as well as to understand how the SIRS environment can affect the MSCs behaviour to maximize the response.^28^

The lack of major histocompatibility complex (MHC) class II (HLA‐DR) in baseline MSCs and a low expression of MHC class I membrane proteins favour the low immunogenicity attributed to this cell type.^29^, ^30^ Furthermore, MSCs do not express co‐stimulatory proteins (CD40, CD80 and CD86) that enhance host T‐cell and NK activation.^31^, ^32^ The low immunogenicity of MSCs favours the use of allogeneic cells without the need for HLA compatibility, and this has virtually no capacity for clinically relevant immune rejection.^24^

It was not only tested in pre‐clinical models (in vitro and animal models), but also in hundreds of clinical trials in diseases such as GVHD, diabetes, CD, rheumatoid arthritis, osteoarthritis and other inflammatory disorders.^33^, ^34^, ^35^ The promising results obtained in these diseases have opened the MSCs’ clinical evaluation in new areas.^36^, ^37^

Although more than 3000 patients have been included in clinical trials with MSCs and safety regarding neoplastic transformation of the administered cells has not been reported,^38^ the potential risk of MSCs tumourigenic transformation should be minimized to ensure safety.^39^ This can be accomplished through assessing karyotype or performing cGH arrays before releasing cell product batches and using cells in early passages in culture to minimize genetic instability.^39^

## 4 MSCS IMMUNOMODULATORY POTENTIAL IN SIRS

**FIGURE 1:**
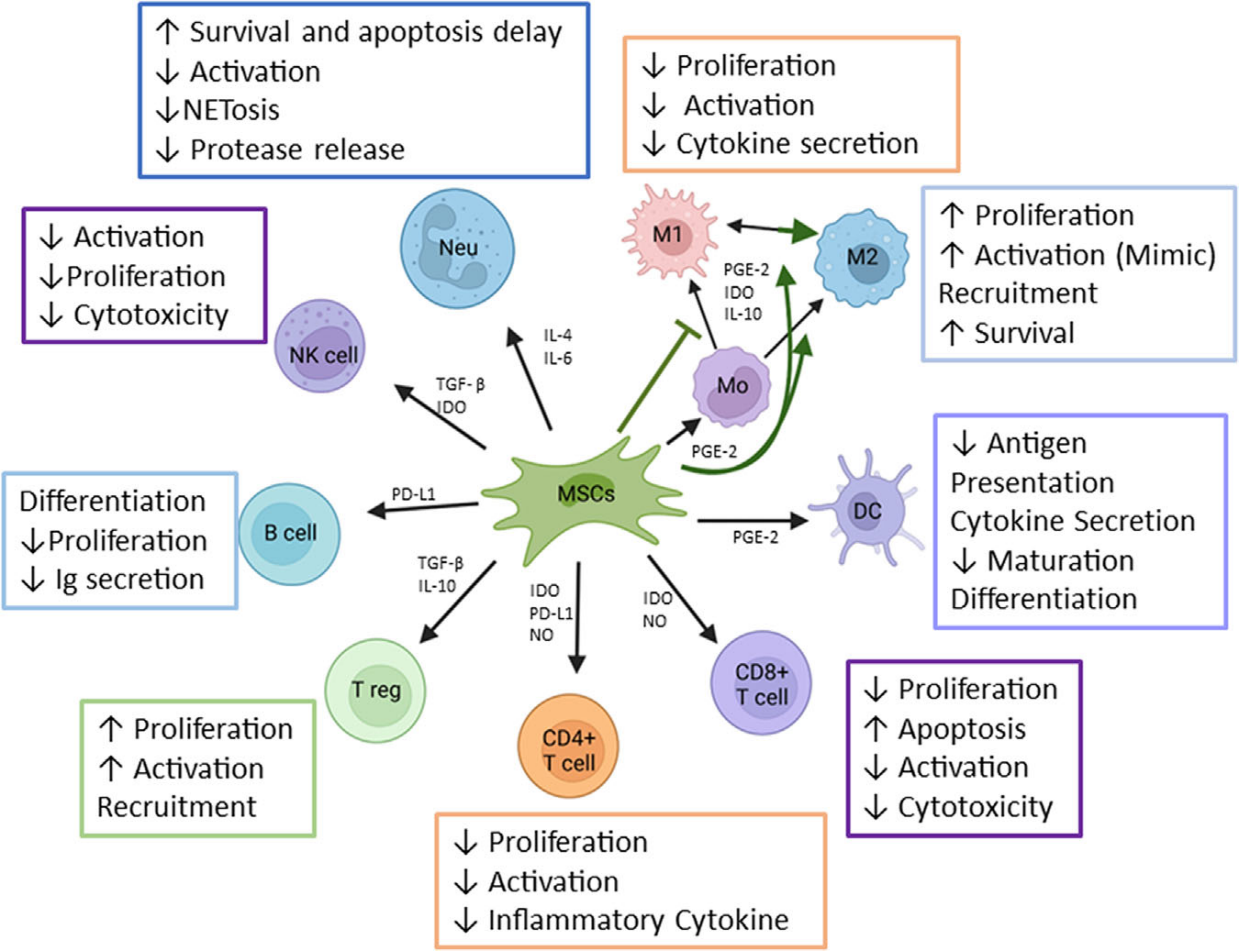
Immunoregulatory activity of mesenchymal stem cells during local and systemic inflammation. Mesenchymal stromal/stem cells (MSCs) interact with all broad of immune cells inducting them to an anti‐inflammatory condition or blocking their pro‐inflammatory activity. MSCs regulate immune cells through direct cell‐to‐cell contact, secretion of cytokines, extracellular vesicles, mitochondrial transference and efferocytosis favouring inflammation reduction. DC, dendritic cell; Ig, immunoglobulin; M1, macrophage type 1; M2, macrophage type 2; Mo, monocyte; Neu, neutrophil; NK, natural killer cell; T reg, regulatory T cell

In inflammatory conditions, MSCs migrate to damaged tissues responding to signalling factors such as DAMPs and chemokines.^40^, ^41^ Both tissue‐resident and mobilized MSCs respond by promoting an anti‐inflammatory state.^42^, ^43^ MSCs induce the restoration of local and systemic homeostasis by secreting, among others, prostaglandin E‐2 (PGE‐2), tumor growth factor‐β (TGF‐β), indoleamine‐2,3‐dioxygenase (IDO), nitric oxide, interleukin‐10 (IL‐10), IL‐4 and chemokines (CCL‐2 and CCL‐5) (Figure 1).^44^, ^45^ Furthermore, MSCs interact with immune cells by several mechanisms, including direct contact (PD‐L1, PD‐L2, CD54/ICAM, CD106/VCAM) or mitochondrial transfer.^46^ Through these mechanisms, MSCs diminish immune cell proliferation and activation, reducing the release of pro‐inflammatory molecules.^47^ A more detailed description of these MSCs regulatory mechanisms over specific immune cells is described later. In several conditions that may lead to SIRS, as polytrauma or infections, previous works (including ours), have shown that MSCs are activated by the abnormal microenvironment conditions (e.g. hypoxia, inflammatory and apoptotic signals), adapting their baseline properties to potentially restore the tissue homeostasis.^48^, ^49^ Activated MSCs can reduce the immune collateral damage by modulating the cytokine storm and immune overactivation.

**FIGURE 2:**
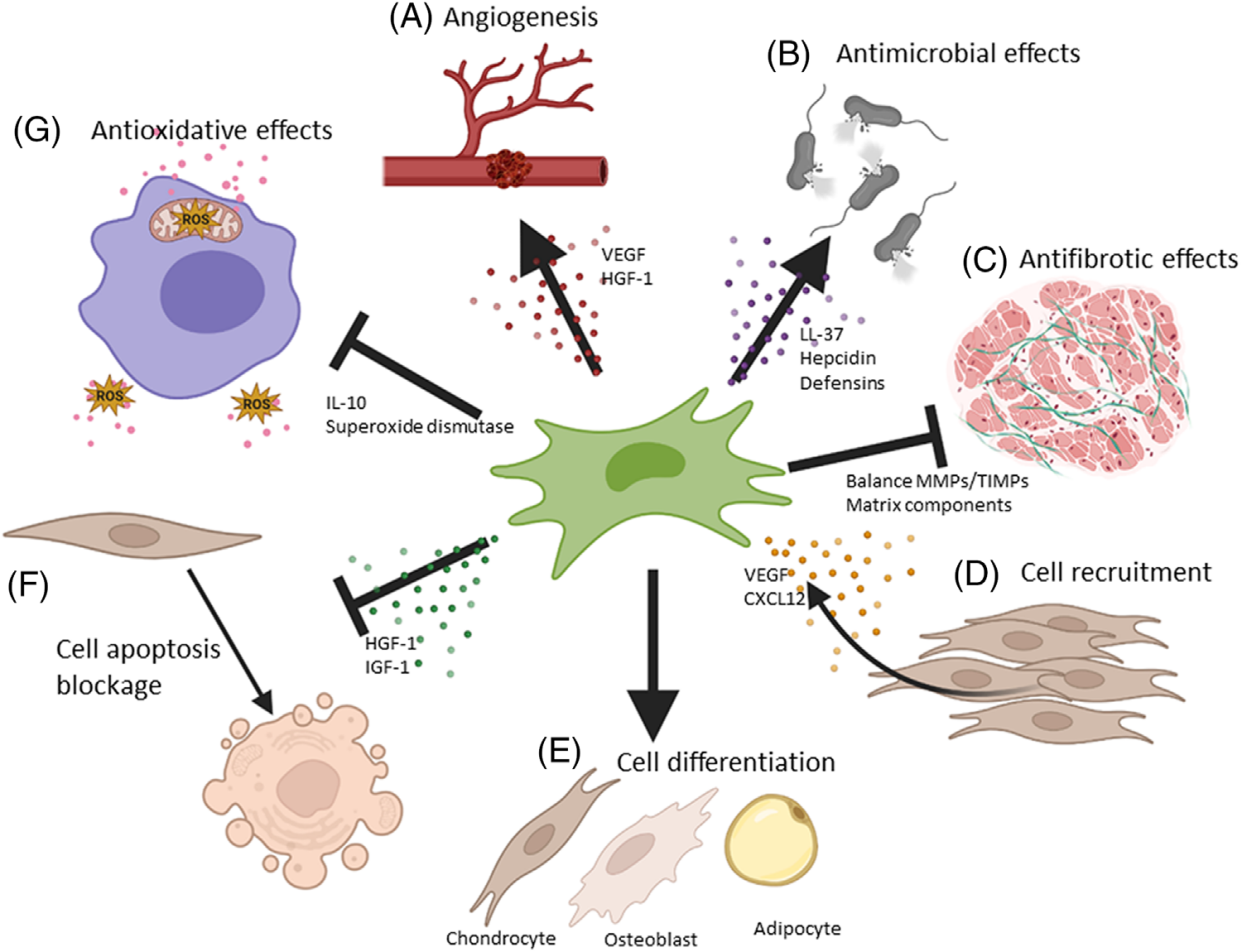
Mesenchymal stem cell properties for tissue protection and regeneration. Mesenchymal stromal/stem cells (MSCs) under stress conditions (e.g. hypoxia) promote tissue restorative activities such as (A) pro‐angiogenesis and endothelial protection, (B) antimicrobial effects (C) anti‐fibrosis, (D) cell recruitment and induction of proliferation, (E) cell differentiation, (F) anti‐apoptotic effect and (G) blockage of reactive oxygen species (ROS) and release of cytotoxic products (antioxidant effects).

Although most MSCs administered via i.v. are initially retained in the lung microvasculature, their effect can be attributed to the changes induced on most cells of the immune system and also by the release of cytokines and growth factors (Figures 1 and 2).^50^, ^51^ Besides from secreting multiple regulatory factors (IDO, IL‐10, PGE‐2), MSCs exert systemic immunomodulation by releasing extracellular vesicles (EVs).^47^, ^52^ MSC‐EVs cargo is incorporated by the recipient cell (adjacent or distant cell) and regulate intracellular pathways. MicroRNAs (small non‐coding RNAs) from MSC‐EVs participate in transcriptional regulation inhibiting the translation of specific genes (complementary sequence) in terms of controlling inflammation.^53^

Comparing to direct MSCs therapy, the infusion of MSC‐EVs may have some advantages. MSC‐EVs are not able to self‐replicate. EVs are stable and can easily be storage for long periods.^54^ Furthermore, due to their small size can cross extracellular matrix and barriers (including haemato–encephalic barrier) reaching target tissues. However, on a functional and clinical level, EVs have not demonstrated to reach the therapeutic activity of MSCs. This may be due to the fact that MSCs do not only act via their EVs, but also through direct cell‐to‐cell contact and by secreting other factors.^55^ Nevertheless, these encouraging results stimulate the need to improve EVs knowledge (cargo comprehension, isolation/purification techniques improvement, normalization following MISEV2018 guidelines, scaling‐up production) and doses strategies to improve their therapeutic potential.

MSCs have shown a protective role against activated neutrophils under inflammatory conditions.^56^ Neutrophils are very susceptible to microenvironmental changes undergoing a rapid activation that induces the release of cytotoxic and anti‐microbial factors (proteases, ROS and neutrophil extracellular traps – NETs) with tissue‐cell detrimental effects before undergoing apoptosis.^57^ This massive cell death induces the release of high amounts of cytotoxic products that seriously impact the surrounding tissues.^56^ MSCs not only decrease neutrophil‐derived ROS, the release of matrix metalloproteinases (MMPs) and NET production but also reduce considerably neutrophil apoptosis.^58^, ^59^ MSCs protect tissues indirectly from overactivated neutrophils by suppressing their activity and avoiding a disproportionate acute death‐cytotoxic release. Through different endogenous antioxidant mechanisms such as the release of superoxide dismutase and glutathione peroxidase (antioxidant enzymes), MSCs reduce oxidative stress and tissue instability.^60^

Local and circulating monocytes migrate towards the inflammatory site and differentiate into pro‐inflammatory macrophages (M1). These phagocytic and antigen‐presenting cells increase the local instability by secreting inflammatory molecules, ROS and proteases. MSCs inhibit the differentiation of monocytes into M1‐macrophages promoting the switch towards the M2 (anti‐inflammatory) phenotype by the secretion of anti‐inflammatory factors including IL‐10, IDO and PGE‐2^61^, ^62^ or by inducing their efferocytosis.^63^, ^64^ MSCs can modulate macrophagic response through these mechanisms, stimulating their production of IL‐10 and TGF‐β.^62^, ^65^ This secondary combined cytokine production wave potentiates a faster shift towards an anti‐inflammatory microenvironment favouring homeostasis and reducing tissue damage.^52^

Dendritic cells (DCs) as major antigen presenting population play a crucial role in the activation and the transition from the innate to the adaptative immune response. Through the MHC antigen presentation, DCs induce the activation and differentiation of T cells.^66^ MSCs can migrate throughout the body and reach secondary lymph nodes suppressing DCs maturation and the subsequent T cell activation.^67^ MSCs and MSC‐EVs modify the cytokine expression pattern of DCs promoting the expression of IL‐10, IL‐6 and TGF‐β, and decreasing IFN‐γ production (promoting a tolerogenic DC phenotype).^68^ Regarding the effects on cells of the immune system, MSCs and MSC‐EVs modify the cytokine expression pattern of DCs promoting the expression of IL‐10, IL‐6 and TGF‐β, and decreasing IFN‐γ production (shift to a tolerogenic DC phenotype).^68^

Although the adaptative response is not as relevant as the innate immune system in the acute phase of SIRS, it has a crucial role in its progression and resolution. Both T and NK cells participate in the production of pro‐inflammatory molecules (TNF‐α, IFN‐γ) and cytolytic (cytotoxic) products.^69^, ^70^ Activated NK cells are known to produce high levels of IFN‐γ and possess high cytolytic activity (perforin and granzyme) that may induce tissue damage in SIRS and sepsis.^71^ Through IDO expression (and the consequent tryptophan metabolism change) and PGE‐2 production, MSCs inhibit the proliferation, the cytotoxicity activity and the cytokine production of NK cells.^72^

In addition to producing antibodies, activated B‐cells release cytokines that regulate immune responses. As a result, B‐cells play a decisive role in the pathogenesis and the outcome of immune disorders. Regarding their action on B‐cells, MSCs can modulate B‐cell activation and differentiation through cell‐to‐cell contact, cytokine and EVs, promoting an immunosuppressive activity (B‐cells increase the production of IL‐10 and TGF‐β).^73^

MSCs can induce T lymphocyte inactivity inhibiting their first activation functions (pro‐inflammatory cytokine release and proliferation) through blocking the CD28 phosphorylation and by reducing the co‐stimulatory antigen presentation by the blockage of DC differentiation.^74^ Moreover, MSCs are known to block lymphocyte proliferation by secreting IDO, TGF‐β, IL‐10 and stabilizing pro‐inflammatory circulating cytokines after interacting with different activated immune cells.^44^, ^75^ Another contributing mechanism to control inflammatory conditions is to promote T regulatory cells (Tregs) differentiation. MSCs activate Tregs by cell contact and cytokine secretion (IL‐10, TGF‐β), increasing Treg circulating levels during sepsis, showing higher levels of anti‐inflammatory cytokines.^76^ Furthermore, MSC‐VEs modulate lymphocytes reducing their IFN‐γ production, metabolism and increasing their expression of Foxp3 (inducing Treg expansion).^77^ In addition to the M2 macrophage induction, MSCs through increasing Tregs can generate an enhanced and durable anti‐inflammatory response.^62^, ^78^ All these immunoregulatory mechanisms may reduce the overactivation of the immune system during SIRS.

## 5 POTENTIAL MECHANISMS FOR TISSUE PROTECTION AND REGENERATION IN SIRS BY MSCS

In addition to their immunomodulatory activity, MSCs have shown regenerative properties (Figure 2).^21^ Resident and mobilized MSCs secret proangiogenic growth factors as VEGF (vascular endothelial growth factor) and HGF‐1 (hepatocyte growth factor) via the HIF‐1 signalling pathway, which promote blood vessel formation and cell recruitment,^79^, ^80^ improving tissue oxygenation. Furthermore, VEGF and HGF protect endothelial integrity from oxidative (ROS) and reduce apoptosis,^81^ which further prevents immune cell transendothelial migration into unspecific tissues.

MSCs may favour some anti‐microbial activity against a several number of pathogens through the secretion of peptides such as LL‐37 (cathelicidin family), hepcidin or defensins. These are directed against pathogen structures or favour immune system recognition, cell recruitment or an increase in macrophage and neutrophil phagocytosis activity.^82^, ^83^ This MSCs property is especially interesting in SIRS and sepsis.

During SIRS, many pro‐apoptotic factors are released into the microenvironment by necrotic and inductor cells. MSCs may also promote local cell survival by the secretion of anti‐apoptotic and healing molecules, including HGF‐1 and insulin‐like growth factor‐1.^41^ MSC‐EVs exert protective cell protection through miRNAs transference. For example, miR‐223 contained in MSC‐EVs showed protection against cardiac dysfunction, apoptosis and inflammatory response.^84^

The repair process after hyper‐inflammation is crucial to prevent or reduce long‐term organ failure.^8^ A rapid and abnormal tissue repair process can lead to irreversible scarring, resulting in organ and tissue fibrosis and dysfunction (hepatic cirrhosis, pulmonary fibrosis etc.). MSCs exert antifibrotic activity by controlling extracellular matrix reorganization, fibroblast secretory activity and the balance between MMPs and their inhibitors.^85^, ^86^

Finally, it has to be stressed that MSCs can also contribute to tissue repair by direct differentiation into mesodermal tissue cells such as osteocytes, adipocytes, chondrocytes, myoblast and perivascular cells.^21^, ^87^ In addition, during SIRS, MSCs may migrate and replace damaged or necrotic cells contributing to tissue regeneration. All these tissue protection and repair capacities of MSCs may prevent major organ lesions and long‐term sequelae in this setting.

## 6 PRE‐CLINICAL AND CLINICAL USE OF MSCS IN ACUTE HYPER‐INFLAMMATORY DISEASES

With the current knowledge of MSCs activity in inflammatory and immune‐based diseases, there is rational for their use in those entities with an acute hyper‐inflammatory phase as polytrauma or infection, two conditions commonly leading to SIRS.

Several pre‐clinical models have shown the potential benefit of MSCs in this setting. For instance, after cecal ligation, the induced bacteriaemia and endotoxin (LPS)‐mediated sepsis can be controlled by MSC infusion, increasing survival in treated animals.^88^ MSCs diminished the systemic inflammatory reaction by reducing pro‐inflammatory cytokine levels (TNF‐α, IL‐1β, IFN‐γ, IL‐1α, IL‐6) while increasing IL‐4 and IL‐10.^89^ In a radiation‐induced MODS non‐human primate model, MSCs showed an enduring distribution across affected organs, reducing the severity of the lesions and increasing the regeneration of damaged tissues.^90^ It is well known that most the intravenously infused MSCs (up to 80%) are initially retained in lung microvasculature.^91^, ^92^ Ortiz et al. demonstrated that MSCs ameliorate lung inflammation and fibrosis in a severe injury lung murine model.^93^ In pre‐clinical models of ARDS MSCs infusion induced a higher bacterial clearance in damaged lungs compared to untreated animals.^94^, ^95^ Curley et al. demonstrated that MSCs restore lung function decreasing inflammation, and reducing reactive immune cells and pro‐inflammatory cytokines in the alveoli.^96^ In this regard, levels of neutrophils and pro‐inflammatory cytokines present in bronchoalveolar lavage samples diminish after MSCs intravenous infusion.^97^ Furthermore, MSCs have shown synergistic effects with antibiotic therapy enhancing bacterial clearance (peritoneal fluid and blood), reducing organ damage and controlling the inflammatory response during sepsis and increasing overall survival in a murine model of sepsis.^98^

The combination of MSCs with other immune‐effector cells, as Tregs, has shown potential synergistic effects with higher reduction in the neurological and systemic inflammation compared to MSCs alone in a brain trauma animal model.^99^ Finally, MSC activation (e.g. through priming with pro‐inflammatory cytokines) or genetic modification (e.g. overexpression of HIF‐1‐alpha, CXCR4 and IL‐10)^100^, ^101^ may enhance their immunomodulatory potential.^102^

Besides the systemic effects and the local effects in the lungs, MSCs infusion has shown favourable results in other SIRS‐affected organs (e.g. heart and kidney) decreasing inflammatory and organ dysfunction biomarkers. Several pre‐clinical models described the potential benefits of MSCs after myocardial injury, showing an increase in cardiac repair and neo‐angiogenesis.^103^ In a kidney injury model, animals treated with MSCs displayed decreased serum creatinine, blood urea nitrogen levels and improved their overall survival.^92^, ^104^ In the same way, hepatic function was restored by MSCs in a liver fibrosis model, reducing inflammation.^105^ Besides the liver, MSCs have shown to prevent fibrosis in organs such as lungs, kidneys and heart.^85^ Results in sepsis need to be clarified in future studies, as there are favourable results and others where no benefit is observed. For instance, in a peritonitis‐induced sepsis porcine model, no major changes in sepsis parameters were observed after MSCs administration. Neither haemodynamic variables nor SOFA (sequential organ failure assessment) scores were different from those of the control group.^106^ Nevertheless, in a different work, MSC infusion showed a significant improvement in animal outcome after sepsis, with especial benefit in cardiovascular function together with a significant reduction in serum lactate levels and vasopressor requirements.^107^ Comparing these last two studies, there are some differences that may influence the final outcome observed. This includes the source of MSCs (UC vs. BM) and the sepsis induction model.^106^

To date, the number of clinical trials that have addressed the role of MSCs therapy in SIRS/sepsis patients is scarce. A search in the clinical trials NIH database (https://clinicaltrials.gov/, accessed September 2022) searching for phases I to III clinical trials with MSCs for the treatment of SIRS or sepsis yielded only the trials indicated in Table 1.

**TABLE 1:** Clinical trials of mesenchymal stem cell (MSC) therapy for systemic inflammatory response syndrome (SIRS)

Identifier	Phase	Condition	MSCs dose and source	Infusion	Subjects	Ref.
NCT02421484	Phase I	Septic shock	0.5 × 10^6^, 1 × 10^6^, or 3 × 10^6^ cells/kg of allogenic BM‐MSCs	Intravenous; one dose	Nine patients; Observational cohort with 21 patients	^36^, ^113^
NCT02328612	Phase I	*Escherichia coli* LPS intravenous infusion (2 ng/kg for 1 min)	.25 × 10^6^, 1 × 10^6^, or 4 × 10^6^ cells/kg of allogenic AT‐MSCs	Intravenous, one dose	32 healthy subjects	^108^
NCT01849237	Phase I	Septic shock with neutropenia	1 × 10^6^ of allogenic BM‐MSCs/kg	Intravenous; one dose	15 patients with CT and MSCs therapy	^114^
NCT05283317	Phase I	Sepsis and septic shock	1 × 10^6^/kg of allogenic AT‐MSCs, on the first, third, fifth, seventh and ninth of allogenic AT‐MSCs	Intravenous; five doses	10 patients with CT and MSCs therapy. Observational cohort of 20 patients (control)	^115^
NCT 04961658	Phase I	Septic shock	15 × 10^6^, 60 × 10^6^ or 150 × 10^6^	Intravenous; one dose	21 patients	Ongoing
NCT 03369275	Phase II	Septic shock	300 × 10^6^ allogenic BM‐MSCs	Intravenous; one dose	114 patients	Ongoing

Abbreviations: AT‐MSCs, adipose‐derived mesenchymal stem/stromal cells; BM‐MSCs, bone marrow‐derived mesenchymal stem/stromal cells; CT, conventional therapy; LPS, lipopolysaccharide.

In a lipopolysaccharide‐induced host response study in 32 healthy human volunteers, three different groups of doses of adipose‐derived MSCs (or placebo) were established. MSCs were infused 1 h before LPS (2 ng/kg for 1 min) administration (NCT02328612). C‐reactive protein levels and flu symptoms did not differ between groups, but the number of neutrophils increased in relation to MSCs dosage, whereas degranulation markers did not change among groups. MSCs promote neutrophil survival by partially reducing the release of cytotoxic products and increasing IL‐10, TGF‐β and IL‐8 plasma concentrations after peaking at 2 h.^108^ It has been described that some subjects may display changes that may favour a pro‐coagulant milieu after MSCs infusion and LPS administration.^108^ It should be noticed that MSCs overexpress, among others, tissue factor (CD142).^28^, ^109^ This fact is most important in the setting of SIRS, and careful evaluation of the route of cell administration (especially from some sources as adipose tissue or placenta) and pro‐coagulant activity should be evaluated in future trials.^28^, ^110^, ^111^ Moreover, if the clinical product is cryopreserved and thawed immediately before infusion, an additional increase in pro‐coagulant activity of MSCs has been suggested, and special attention should be given.^111^, ^112^

In another study, MSCs infusion, with doses up to 250 million cells, was well tolerated and safe in septic shock patients (NCT02421484), with no adverse events and differences in SOFA score, hospital stay or mortality.^113^ Following the administration of MSCs to nine septic patients, levels of plasma pro‐inflammatory cytokines decreased in a dose‐specific manner compared to an observational cohort group.^36^ Based on these results, the same group has recently launched a new phase II clinical trial for the treatment of septic patients with allogenic BM‐derived MSCs (NCT03369275). In another clinical trial of 30 patients with advanced sepsis and neutropenia, MSCs therapy (along with conventional treatment) increased short‐term survival attenuating the shock‐related organ dysfunction. However, no differences in overall survival at day 28 were observed because patients remained immunocompromised being the infection the cause of death in most cases.^114^ The role of multiple sequential doses has also been assessed. A recent study proposed the administration of four sequential doses of 1 × 10^6^/kg after the first infusion to extend the effects of MSCs therapy. MSCs administration showed significant benefits in the early phase (higher surveillance) with the decrease of SOFA score. No adverse events were observed during MSCs infusion in 10 treated patients.^115^ Finally, the role of dose intensity is also being tested in phase I clinical trial (NCT04961658) that will evaluate the dose efficacy (15 × 10^6^, 60 × 10^6^ or 150 × 10^6^) in septic shock patients.

## 7 PROSPECTS OF MSC THERAPY FOR SIRS AND FINAL COMMENTS

With the early evidence of the potential benefit of MSC therapy for SIRS patients, and if the current phase I/II clinical trials (see Table 1) show evidence of clinical efficacy, we expect to see the conduct of phase III clinical trials in the upcoming years. In addition to standard MSCs production, different strategies (pre‐conditioning, cytokine or hypoxic priming) have been proposed to optimize MSCs therapy (increasing their homing, immunomodulatory capacities, paracrine production, lifespan and more durable effects).^102^, ^116^ Genetic modification has also been shown to enhance MSCs therapy for a higher production of regulatory factors or repair capacity.^116^ Some of these strategies are also under clinical evaluation. As an alternative to MSCs, the use of MSC‐EVs has also been proposed for SIRS treatment due to a number of potential and regulatory advantages.^117^ Prospects of the future research of MSCs therapy in SIRS patients include the evaluation of the direct effects of MSCs activation by SIRS inflammatory molecules and the role of treatment combinations on MSCs function. Future research on MSCs therapy for SIRS patients should include the evaluation of the optimal timing for cell administration, and the selection of the optimal source, administration route and dosing, including the possibility of sequential doses.

## 8 CONCLUSIONS

In summary, MSCs emerge as a potential alternative for critical patients with SIRS. We suggest that MSCs therapy is a potential tool that could be helpful in modulating innate and adaptive immune system activation, massive inflammatory response and tissue damage in patients with SIRS. However, further studies are required to understand SIRS physiopathology and to adapt an effective strategy of MSCs therapy in the critical‐care unit.

## CONFLICTS OF INTEREST

FSG has received honoraria and/or research support from Celgene/BMS, Kite/Gilead, Novartis, Pfizer, Roche and Takeda, none of them related to the current manuscript. ANB has received honoraria from Abbvie, AstraZeneca and Janssen, none of them related to the current manuscript. The remaining authors declare no conflicts of interest.
